# Human Bone Marrow Adipose Tissue is a Metabolically Active and Insulin-Sensitive Distinct Fat Depot

**DOI:** 10.1210/clinem/dgaa216

**Published:** 2020-04-20

**Authors:** Tam T Pham, Kaisa K Ivaska, Jarna C Hannukainen, Kirsi A Virtanen, Martin E Lidell, Sven Enerbäck, Keijo Mäkelä, Riitta Parkkola, Sauli Piirola, Vesa Oikonen, Pirjo Nuutila, Riku Kiviranta

**Affiliations:** 1 Turku PET Centre, University of Turku, Turku, Finland; 2 Institute of Biomedicine, University of Turku, Turku, Finland; 3 Department of Medical Biochemistry and Cell Biology, Institute of Biomedicine, The Sahlgrenska Academy, University of Gothenburg, Gothenburg, Sweden; 4 Department of Orthopaedics and Traumatology, Turku University Hospital, Turku, Finland; 5 Department of Radiology, Turku University Hospital, Turku, Finland; 6 Department of Endocrinology, Turku University Hospital, Turku, Finland

**Keywords:** bone marrow, adipose tissue, PET/CT

## Abstract

**Context:**

Bone marrow (BM) in adult long bones is rich in adipose tissue, but the functions of BM adipocytes are largely unknown. We set out to elucidate the metabolic and molecular characteristics of BM adipose tissue (BMAT) in humans.

**Objective:**

Our aim was to determine if BMAT is an insulin-sensitive tissue, and whether the insulin sensitivity is altered in obesity or type 2 diabetes (T2DM).

**Design:**

This was a cross-sectional and longitudinal study.

**Setting:**

The study was conducted in a clinical research center.

**Patients or Other Participants:**

Bone marrow adipose tissue glucose uptake (GU) was assessed in 23 morbidly obese subjects (9 with T2DM) and 9 healthy controls with normal body weight. In addition, GU was assessed in another 11 controls during cold exposure. Bone marrow adipose tissue samples for molecular analyses were collected from non-DM patients undergoing knee arthroplasty.

**Intervention(s):**

Obese subjects were assessed before and 6 months after bariatric surgery and controls at 1 time point.

**Main Outcome Measure:**

We used positron emission tomography imaging with 2-[^18^F]fluoro-2-deoxy-D-glucose tracer to characterize GU in femoral and vertebral BMAT. Bone marrow adipose tissue molecular profile was assessed using quantitative RT-PCR.

**Results:**

Insulin enhances GU in human BMAT. Femoral BMAT insulin sensitivity was impaired in obese patients with T2DM compared to controls, but it improved after bariatric surgery. Furthermore, gene expression analysis revealed that BMAT was distinct from brown and white adipose tissue.

**Conclusions:**

Bone marrow adipose tissue is a metabolically active, insulin-sensitive and molecularly distinct fat depot that may play a role in whole body energy metabolism.

Adipose tissue has emerged as a more heterogeneous tissue than previously appreciated. The different metabolic and prognostic characteristics of visceral and subcutaneous fat depots are well characterized, while we and others have also shown that functional brown adipose tissue (BAT) is present in adults and that the tissue may have significant metabolic functions (1, 2). Moreover, newly discovered inducible brown fat cells, or beige adipocytes, appear to be molecularly distinct from both white and brown adipocytes (3, 4).

Bone marrow adipose tissue (BMAT) has been hypothesized to be functionally distinct from both white and brown adipose tissue and to contribute to systemic and skeletal metabolism (5–7). At birth, the bone marrow (BM) cavity of long bones is filled with hematopoietic bone marrow that later, during growth, persists mainly in the BM spaces of flat bones such as sternum and ilium (8). The majority of the BM space in adult long bones is filled with BMAT. The total amount of BMAT in an average person is estimated to be 0.5 to 3 kg and accounting for approximately 8% of total fat mass in the average-sized human. Depending on the peripheral fat volume, it could account up to 30% of total body fat (6). Despite its wide presence, the functions of BMAT in normal energy metabolism, bone turnover, and in disease are largely unknown. Previous studies have shown that the amount of BMAT increases with age and correlates with the amount of visceral fat in obese women (9). The amount of BMAT is also increased during caloric restriction (10), unloading of the skeleton (11), and in several diseases, such as anorexia nervosa (12–14) and upon the failure of hematopoietic BM (15). Mouse studies have suggested that BMAT may have some features of BAT (16), but this has not been reported in humans. BMAT at least in rodents may also be more heterogeneous and exist as both constitutive and regulated marrow adipose tissues (17).

We aimed to demonstrate that BMAT is a metabolically and molecularly distinct fat depot, differing from the white, brown, and beige adipose tissues. For these purposes we characterized BMAT using positron emission tomography/computed tomography (PET/CT) imaging with 2-[^18^F]fluoro-2-deoxy-D-glucose ([^18^F]-FDG) tracer and molecular profiling. We investigated whether (1) BMAT is an insulin-sensitive tissue, and (2) if the level of insulin sensitivity is altered in morbidly obese subjects and in patients with type 2 diabetes (T2DM). Furthermore, we tested if bariatric surgery could modulate the glucose metabolism of BMAT.

## Materials and Methods

### Study subjects and designs

A total of 23 morbidly obese subjects (F/M = 19/4, age = 47.0+/-1.9 yrs, BMI = 43.1+/-0.8) and 9 healthy subjects with normal body weight (F/M = 7/2, age = 46.8+/-2.0 yrs, BMI = 23.7+/-0.7) were recruited to study GU in BMAT. Obese subjects were randomized to bariatric surgery either by gastric bypass (N = 13) or sleeve gastrectomy (N = 10). Nine of the obese subjects had T2DM according to the American Diabetes Association criteria and 5 were prediabetic with either impaired fasting glucose (IFG, n = 1), impaired glucose tolerance (IGT, n = 2), or both (n = 2). In the data analysis patients with IFG and/or IGT were classified as diabetics. As described earlier (18), 4 patients were newly diagnosed with T2DM based on the results of the oral glucose tolerance test (OGTT) at the screening and were treated with metformin. In the other 5 patients, the median diabetes duration was 2.8 years. They were treated with oral antidiabetic drugs (2 with metformin; 1 with metformin/sulphonylurea/gliptin; 1 with metformin/pioglitazone/gliptin, and 1 with metformin/pioglitazone). The medications were paused before positron emission tomography (PET) studies as follows: pioglitazone 3 days before and other medications 1 day before PET imaging. None were on insulin treatment. The inclusion and exclusion criteria have been described previously (19, 20). Anthropometry, including body height, body weight, waist, and hip circumferences, was collected at a screening visit before inclusion to the study and 6 months after the operation, and a standard 2-hour OGTT after an overnight fast was performed at both study visits. Glucose uptake was analysed using 2-[^18^F]fluoro-2-deoxy-D-glucose ([^18^F]-FDG) and PET. Glucose uptake in vertebral bone marrow, femoral bone marrow, muscle, subcutaneous fat, and visceral fat was analyzed at baseline visit and in morbidly obese subjects also 6 months after the surgery. At both visits, the [^18^F]-FDG-PET study was performed at fasting state and during hyperinsulinemic (40mU/m^2^ per min) euglycemic (5 mmol/L) clamp (21). Whole body insulin sensitivity was calculated as glucose disposal rate (M value; µmol/kg/min) during the last hour of the clamp. The insulin-resistance index was determined by the homeostasis model assessment (HOMA-IR) as calculated as the product of fasting serum insulin (mU/L) and fasting plasma glucose (mmol/L) divided by 22.5.

To analyze the response of BMAT to cold exposure, we recruited 11 healthy volunteers (F/M = 8/3, age = 40.6+/-2.8, BMI = 23.1+/-0.9). Glucose uptake was assessed in vertebral bone marrow, humeral bone marrow, and skeletal muscle in the arm. Subjects were analysed on 2 consecutive days: on the first day at warm conditions (+23°C) and on the second day under cold conditions (+17°C). On the cold exposure day, the subjects spent 2 hours wearing light clothes in a room with an ambient temperature of 17^ο^C (± 1^ο^C) before moving into the PET/CT room, which has a temperature of +23^ο^C. During PET imaging, 1 foot was placed intermittently (5 minutes in, 5 minutes out) in a cold water bath (+8°C ± 1^ο^C). All analyses were performed after an overnight fast.

The summary of study subjects is provided in Table 1. Study protocols were approved by the Ethical Committee of the Hospital District of Southwestern Finland and written informed consent was given by all study participants before participation.

**Table 1.** Characteristics of the study subjects. Obese subjects were analyzed before bariatric surgery (pre) and six months after bariatric surgery (post) (20). Control and subjects for cold exposure study were analyzed at one timepoint (2)

	Study 1							Study 2
	Control	Obese Non-T2DM			Obese T2DM			Cold
		Pre	Post	Change, %	Pre	Post	Change, %	
N (F/M)	9 (7/2)	14 (13/1)			9 (6/3)			11 (8/3)
Age	46.8 (2.0)	43.1 (2.5)			53.2 (1.5)			40.6 (2.8)
Weight, kg	69.5 (2.3)	120.7 (3.1)	93.7 (3.3)^***^	-22.4 (1.6)	121.5 (3.1)	92.5 (4.9)^***^	-24.1 (2.3)	64.6 (4.1)
BMI, kg/m^2^	23.7 (0.7)	44.1 (1.1)	34.3 (1.1)^***^	-22.2 (1.7)	41.5 (0.8)	31.5 (1.2)^***^	-24.1 (2.3)	23.1 (0.9)
waist-to-hip ratio	0.84 (0.04)	0.89 (0.02)	0.88 (0.02)	-0.9 (1.7)	0.95 (0.04)	0.92 (0.04)	-2.9 (1.7)	0.83 (0.03)
SAT, kg	5.1 (0.6)	21.3 (1.1)	14.4 (1.0)^**^	-33.3 (3.4)	17.8 (1.3)	10.9 (1.1)^*^	-39.8 (2.3)	n/a
VAT, kg	1.8 (0.3)	3.7 (0.4)	2.2 (0.3)^**^	-40.5 (3.7)	4.6 (0.3)	2.8 (0.4)^*^	-40.4 (8.7)	n/a
fP-Glucose, mmol/L	5.4 (0.1)	5.3 (0.4)	5.2 (0.1)	-8.0 (2.3)	7.1 (0.7)	5.8 (0.2)^*^	-15.7 (5.2)	5.2 (0.1)
HbA1c, %	5.7 (0.1)	5.6 (0.1)	5.5 (0.1)^*^	-2.7 (1.0)	6.5 (0.2)	5.8 (0.1)^**^	-9.7 (3.4)	n/a
HOMA index	1.5 (0.3)	4.1 (0.6)	1.3 (0.2)^**^	-62.7 (8.0)	8.1 (3.6)	2.0 (0.3)^**^	-60.3 (6.5)	1.2 (0.3)
M value, µmol/min/kg	41.0 (3.3)	13.0 (1.6)	26.0 (2.8)^**^	102.8 (19.1)	11.9 (1.9)	20.9 (2.4)^*^	100.4 (32.9)	49.1 (6.9)
CTX, ng/mL	0.37 (0.07)	0.30 (0.05)	0.70 (0.07)^**^	216.2 (57.0)	0.24 (0.06)	0.81 (0.17)^*^	381.6 (119.1)	n/a
PINP, ng/mL	35,7 (4.8)	40.0 (5.4)	68.1 (6.1)^*^	101.6 (24.7)	36.6 (5.9)	67.6 (8.9)^*^	134.5 (54.2)	n/a
Osteocalcin, ng/mL	7.3 (0.5)	5.0 (0.5)	8.9 (0.7)^**^	91.7 (15.2)	4.5 (0.6)	7.9 (1.2)^*^	101.6 (38.5)	n/a

Data are presented as mean (SEM). Postvalues have been compared to prevalues and statistically significant *P*-values for Wilcoxon test are shown (^***^P < 0.001, ^**^P < 0.01, *P<0.05). Obese non-T2DM group and obese T2DM group did not differ from each other preoperatively (Mann–Whitney U test *P* > 0.05) except for age, which was higher in the T2DM group (*P* = 0.016). Significances between controls and morbidly obese are not indicated, as most of the parameters differed.

Abbreviations: HbA1c, glycated hemoglobin; HOMA, homeostatic model assessment; SAT, subcutaneous adipose tissue; VAT, visceral adipose tissue; n/a, not available.

### [^18^F]-FDG-PET

A GE PET machine DiscoveryTM ST System with a resolution of 3.75 was used for PET studies. Two catheters were inserted, 1 in an antecubital vein for injection of [^18^F]-FDG or insulin, glucose, and [^18^F]-FDG infusions and another in the opposite antecubital arterialized vein for blood sampling. The subjects lied in a supine position throughout the studies. Abdominal subcutaneous and visceral fat, vertebral bone, femoral bone, and skeletal muscle were scanned with [^18^F]-FDG PET. In the cold exposure study, [^18^F]-FDG-PET imaging was performed simultaneously at the upper torso and upper limbs to image GU of BAT (torso) and BMAT (humerus), respectively.

**Figure 1.**
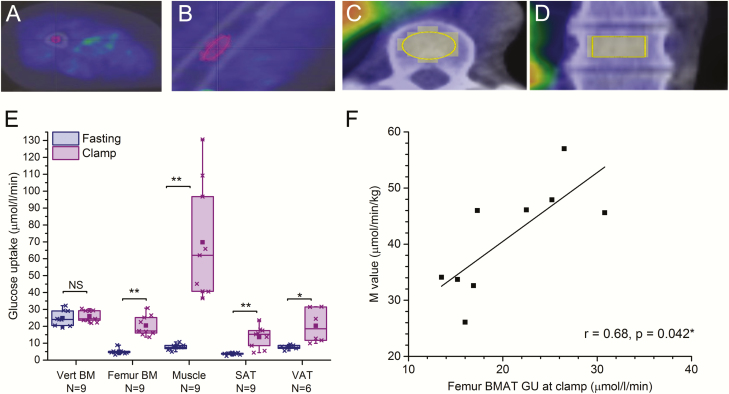
Insulin enhances GU in human femoral BMAT. Glucose uptake (µmol/l/min) in vertebral and femoral BM in healthy control subjects (n = 9). Volume of interest (VOI) in femoral bone marrow in cross-sectional image (**A**) and sagittal image (**B**); and in vertebral bone marrow in cross-sectional image (**C**) and sagittal image (**D**). **E:** Regional GU (µmol/l/min) in lumbar vertebral bone marrow (Vert BM, n = 9), femoral bone marrow (Femur BM, n = 9), skeletal muscle (Muscle, n = 9), abdominal subcutaneous adipose tissue (SAT, n = 9), and visceral adipose tissue (VAT, n = 6) during fasting state and hyperinsulinemic euglycemic clamp. *P*-values for clamp-induced change are given as: *P < 0.05, **P < 0.01, ***P < 0.001, NS not significant *P* > 0.05. The lines of the boxes represent the 25^th^, 50^th^, and 75^th^ percentiles, whiskers 10^th^ and 90^th^ percentiles, and the square indicates the mean value. **F:** Correlation between femur BMAT GU (µmol/l/min) and M-value (µmol/min/kg) (N = 9). Values are mean + SEM.

All image data were corrected for dead-time, decay, and measured photon attenuation. Plasma radioactivity was measured with an automatic gamma counter. Positron emission tomography images were analyzed using CARIMAS 2.6 version. Volumes of interest (VOIs) were manually drawn over the vertebral, humeral, and femoral bone marrow region, avoiding the pixels overlapping the cortical bone area (Fig. 1A–1D), on quadriceps muscle, and abdominal subcutaneous and visceral fat regions. For calculating the uptake of FDG, a 3-compartment model and graphical analysis were employed (22). Plasma and tissue time-activity curves were analyzed graphically to quantify the fractional uptake rate of the tracer (Ki). Glucose uptake in bone marrow was calculated by multiplying Ki with plasma glucose concentration.

### Magnetic resonance imaging

Magnetic resonance imaging (MRI) was used for PET data anatomical reference and for the measurement of abdominal subcutaneous and visceral fat. Data was obtained using 1.5 Tesla system (Intera, Philips Medical Systems, Amsterdam, The Netherlands). Abdominal subcutaneous and visceral adipose tissue volumes (mm^3^) were calculated using SliceOmatic Tomovision software version 4.3 (http://www.tomovision.com/products/sliceomatic.html). Morpho mode and region growing mode were used to semiautomatically draw the VOIs in abdominal subcutaneous and visceral fat, respectively. Adipose tissue mass (kg) was calculated by multiplying adipose tissue volume (mm^3^) with adipose tissue density 0.9196 and divided by 10^6^.

### Biochemical analyses

Plasma concentration of glucose was measured using the glucose oxidase method (Analox GM7 or GM9; Analox Instruments Ltd., London, UK) and serum insulin was determined by time-resolved immunofluorometric assay (AutoDELFIA; PerkinElmer Life and Analytical Sciences , Waltham, MA). Bone resorption was assessed by C-terminal crosslinked telopeptides of type I collagen (CTX, IDS-iSYS CTX-I ELISA, CrossLaps®) and bone formation by intact N-terminal propeptide of type I collagen (PINP, IDS-iSYS Intact PINP assay), both from IDS Ltd., East Boldon, UK. Serum total osteocalcin was determined with 2-site immunoassay based on monoclonal antibodies 2H9 and 6F9 using previously described protocol (23).

### BMAT samples and RNA isolation

Gene expression analysis was performed in BMAT samples harvested from middiaphysis of femur and tibia of 6 patients undergoing knee arthroplasty surgery (F/M = 3/3, age 53.7+/-4.2). Subjects did not have any antidiabetic medication or previously diagnosed diabetes mellitus. The average of femoral and tibial BMAT was used and a sample of subcutaneous white adipose tissue was obtained from the same leg as a reference. Similarly, bone marrow and subcutaneous fat samples were harvested from healthy pigs (N = 6) (24). RNA was extracted from adipose tissue samples using TriSure reagent (Bioline/Meridian Bioscience, Cincinnati, OH) followed by RNA clean-up with RNeasy Mini kit (Qiagen, Hilden, Germany) according to the manufacturers’ protocols, and 58 ng of RNA was reverse transcribed to cDNA using First Strand cDNA Synthesis Kit for RT-PCR (Roche Applied Biosciences, Mannheim, Germany).

### qRT-PCR

For quantitative RT-PCR, we used Power SYBR Green PCR Master Mix and a ViiA7 Real-Time PCR System (both from Applied Biosystems, Waltham, MA). mRNA expression of the following genes was analyzed: peroxisome proliferator-activated receptor gamma (PPARγ), adiponectin (ADIPOQ), insulin receptor (INSR), insulin receptor substrates 1 (IRS1) and 2 (IRS2), and glucose transporter 4 (GLUT4); PR domain containing 16 (PRDM16); beta-3 adrenergic receptor (ADRB3), uncoupling protein-1 (UCP1), T-box protein 1 (TBX1), transmembrane protein 26 (TMEM26), and tumor necrosis factor receptor superfamily member 9 (TNFRSF9, or CD137). Expression levels were normalized to the geometrical mean of the expression of 3-monooxygenase/tryptophan 5-monooxygenase activation protein, zeta (YWHAZ), and 60S acidic ribosomal protein P0 (RPLP0). Primer sequences are available upon request. Gene expression in SAT and BMAT were compared to the gene expression in a well-characterized sample of supraclavicular BAT and expressed as fold compared to the reference BAT sample (set as 1).

### Statistical analyses

Data are presented as mean ± standard errors of the mean (SEM). Normal distribution was assessed using the Shapiro Wilkes test, showing that part of the data was not normally distributed. Wilcoxon signed ranks test was used to compare GU in fasting state and during hyperinsulinemic clamp (insulin stimulation)—and to compare GU in ambient temperature and during cold exposure. Spearman’s test was used to study the correlations between variables. SPSS version 25 was used for statistical analysis and *p* < 0.05 was considered statistically significant.

## Results

### Insulin enhances GU in human femoral BMAT

We first analyzed the regional GU in different fat depots in healthy subjects (N = 9, Table 1). Analysis was performed on vertebral and femoral bone using PET imaging with [^18^F]-FDG tracer at fasting and during hyperinsulinemic euglycemic clamp (Fig. 1A–1D). The highest GU at fasting state was observed in the trabecular bone area of vertebral bodies (*p* < 0.001 compared to other depots analyzed). In vertebrae, GU was not stimulated by insulin (mean fold change ± SEM, 1.1 ± 0.1, *P* = 0.52; Fig. 1E). This region of interest contains multiple cell types, including bone cells, hematopoietic cells, and some adipocytes, and the relative contributions of these to the GU are difficult to determine. To measure GU in an area that consists mainly of BMAT, we analyzed GU in femoral middiaphysis that is mostly filled with bone marrow adipocytes. In contrast to the vertebrae, insulin stimulation increased BMAT GU 4-fold (4.3 ± 0.5, *P* = 0.008), while in subcutaneous adipose tissue (SAT) and visceral adipose tissue (VAT) the increases were 3.5 ± 0.5 (*P* = 0.008) and 2.7 ± 0.6 (*P* = 0.028), respectively (Fig. 1E). As expected, insulin stimulation significantly increased GU in quadriceps muscle nearly 10-fold (9.6 ± 2.2, *P* = 0.008). The level of insulin-induced GU in femur BM was smaller than insulin-stimulated GU in the muscle (*P* = 0.001), but similar to that in SAT (*P* = 0.55) or VAT (*P* = 0.09). Glucose uptake in femur BM positively correlated (r = 0.68, *P* = 0.042) with whole body GU, M-value (Fig. 1F). These data indicate that GU in femoral BMAT can be induced by insulin and demonstrate that BMAT is an insulin-sensitive fat depot.

### Insulin sensitivity of human BMAT is impaired in obesity and type 2 diabetes

**Figure 2.**
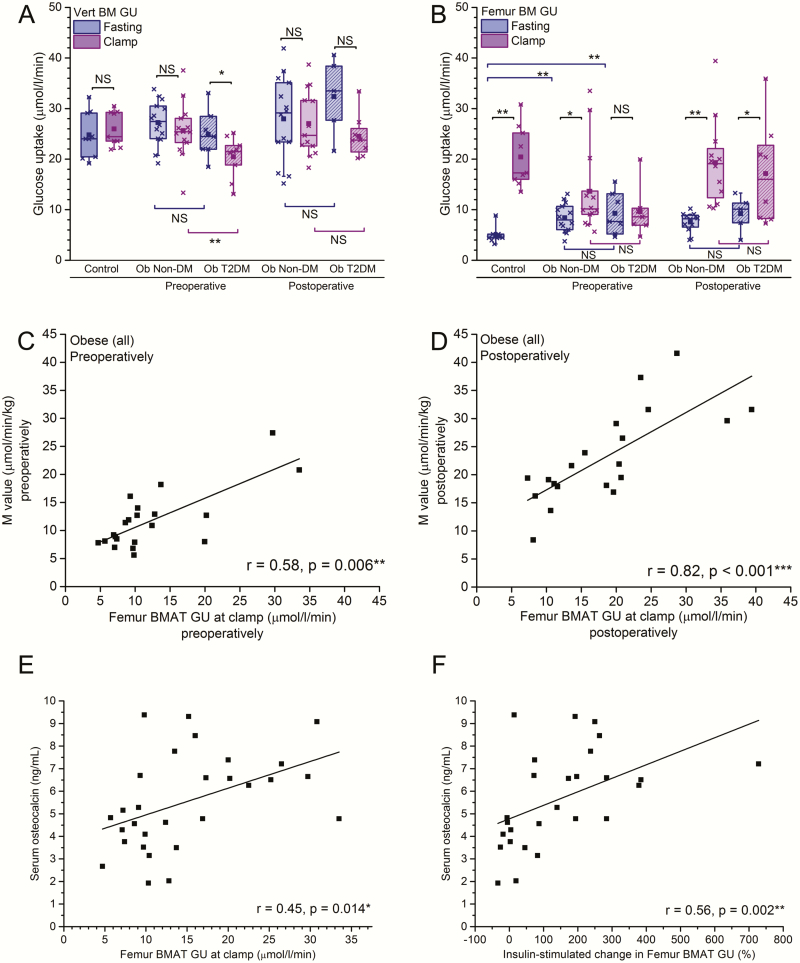
Insulin sensitivity of human BMAT is impaired in obesity and T2DM and can be reversed with weight loss. Vertebral BM GU (µmol/l/min) (**A**) and femur BM GU (**B**) at fast and during hyperinsulinemic clamp in controls (same individuals as in Fig. 1G) and obese subjects (Ob) with or without T2DM. Glucose uptake has been analyzed before bariatric surgery (preoperative) and 6 months after bariatric surgery (postoperative). *P*-values for insulin-induced change are given above the plot *P < 0.05, ** *P* < 0.01, NS not significant *P* > 0.05. In addition, *P*-values for the differences between GU in diabetic and nondiabetic obese subjects are shown below the plot. The lines of the boxes represent the 25^th^, 50^th^, and 75^th^ percentiles, whiskers 10^th^ and 90^th^ percentiles, and the square indicates the mean value. **C:** Correlation between femur BMAT GU (µmol/l/min) and M-value (µmol/min/kg) during hyperinsulinemic clamp before the surgery and after the surgery (**D**).

We next tested whether the GU in BMAT is altered in metabolic disorders, namely morbid obesity with or without T2DM. In vertebrae, GU at fasting was of similar magnitude in obese subjects with or without T2DM and healthy controls (Fig. 2A). As in the controls, insulin stimulation did not affect vertebral bone marrow GU in the obese patients without T2DM, but interestingly there was a modest decrease in the vertebral GU in obese subjects with T2DM (Fig. 2A). In femoral BM, fasting GU was lower in controls than in obese subjects with (*P* = 0.008) or without (*P* = 0.002) T2DM (Fig. 2B). Higher basal GU in obesity could be partially explained by marginally higher insulin concentrations in the obese subjects with T2DM (mean 20.6 mU/L) or without T2DM (15.7 mU/L) when compared to the controls (7.1 mU/L, *P* = 0.06). Insulin stimulation enhanced GU in obese subjects without T2DM (1.7 ± 0.2-fold, *P* = 0.016), although insulin effect was less than in normal weight controls (4.3 ± 0.5-fold, *P* = 0.008). In contrast, in obese subjects with T2DM, there was no difference in BMAT GU at fasting and upon insulin stimulation (*P* = 0.89). This suggests that femoral BMAT is an insulin sensitive tissue, which may become insulin resistant, especially in morbidly obese subjects with T2DM.

To study whether the impaired insulin sensitivity of BMAT could be restored, we analyzed BMAT GU after the morbidly obese subjects had undergone bariatric surgery. As we have reported earlier in this cohort, bariatric surgery resulted in significant decreases in body weight and BMI during the first 6 months, both in obese subjects without T2DM (mean -22%) and with T2DM (mean -24%, both *P* < 0.001) (20, 25). Bariatric surgery also improved the whole body insulin sensitivity, and the M-values approximately doubled (*P* < 0.001) in obese subjects with and without T2DM, and restored the previously suppressed osteocalcin levels close to the control levels (Table 1). Six months after surgery, femoral BM GU increased significantly upon insulin stimulation in obese subjects without T2DM (2.7 ± 0.3-fold, *P* = 0.003), and this increase was more pronounced than before surgery (Fig. 2B). Strikingly, insulin resistance of BMAT observed preoperatively in subjects with T2DM had ameliorated after bariatric surgery and BMAT GU increased significantly upon insulin stimulation also in obese subjects with T2DM (*P* = 0.043). The postoperative basal femoral GU remained, however, higher than in the healthy subjects, probably due to the remaining differences in body weight and insulin sensitivity (Table 1). No changes were seen in vertebral BM GU (Fig. 2A). Femoral BMAT GU during insulin stimulation positively correlated with the whole body insulin sensitivity (M-value) both before (*P *= 0.006, Fig. 2C) and after surgery (*P* < 0.001, Fig. 2D).

### Association between BMAT and bone remodeling

As BMAT has been suggested to interact with the regulation of bone remodeling (26, 27), we investigated if insulin resistance in BMAT would correlate with serum markers of bone turnover. Serum osteocalcin, a marker of osteoblasts and a putative link between skeleton and energy metabolism (28), positively correlated to femoral BMAT GU during insulin stimulation (r = 0.45, *P* = 0.014; Fig. 2E), and to the insulin-induced increase in BMAT GU (r = 0.56, *P* = 0.002; Fig. 2F). We did not observe any association between femoral BMAT GU during insulin stimulation or insulin-stimulated increase in BMAT GU and the markers of bone remodeling, such as CTX, a marker of bone resorption (*P* = 0.84 and *P* = 0.22, respectively), or PINP, a marker of bone formation (*P* = 0.93 and 0.44, respectively). Femur BMAT GU moderately correlated to uCRP in the entire cohort (*P* = 0.047, data not shown) but not in the obese subjects (*P* = 0.91), and there was no association between surgery-induced changes in uCRP and femur BMAT GU (*P* = 0.55, data not shown).

### Human BMAT is not activated by cold exposure and does not express UCP1

**Figure 3.**
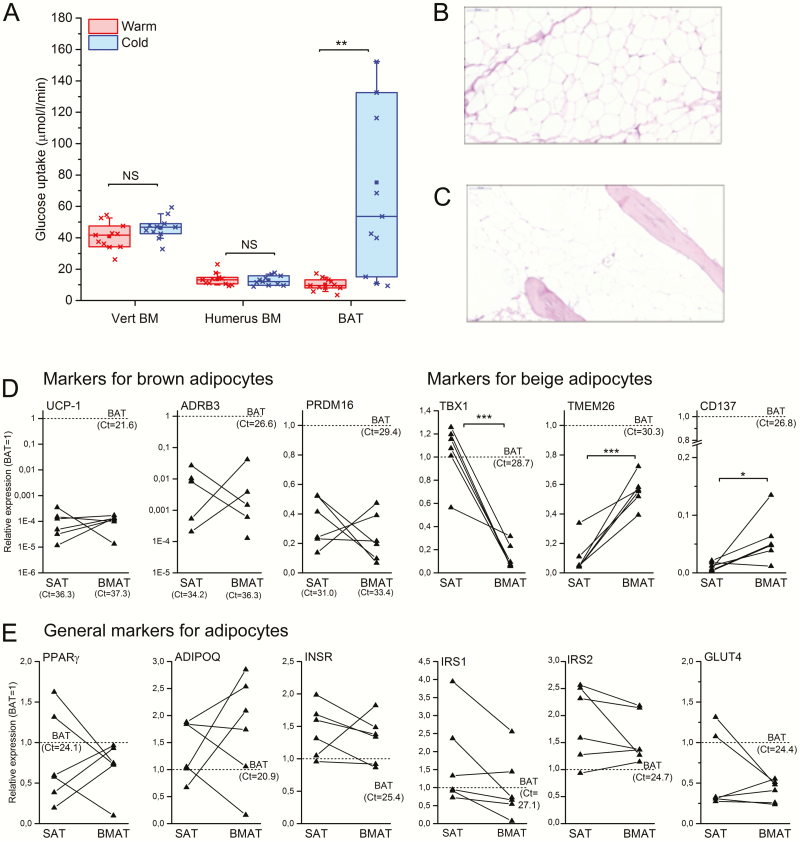
Human BMAT does not have features of brown fat. **A:** Glucose uptake in thoracic vertebral bone marrow (Vert BM), humeral bone marrow (Humerus BM), and subclavicular brown adipose tissue (BAT) at room temperature (warm) and upon cold exposure (cold) (N = 11). *P*-values for cold-induced change are given as: ** *P *< 0.01, NS, not significant difference. The lines of the boxes represent the 25^th^, 50^th^, and 75^th^ percentiles, whiskers 10^th^ and 90^th^ percentiles, and the square indicates the mean value. **B:** Histological section of subcutaneous adipose tissue and C) of long bone BMAT (both 20-fold magnification, hematoxylin-eosin staining). **D–E:** Expression of marker genes for adipocytes in paired human SAT and BMAT samples. Average of femoral and tibia BMAT is used and data is represented as relative expression compared to a reference BAT sample (set as 1, dotted line). Markers for brown adipocytes (UCP1, ADRB3, PRDM16) and beige adipocytes (TBX1, TMEM26M, CD137) (**D**) and brown and white adipocytes (PPARG, ADIPOQ, GLUT4, INSR, IRS1, IRS2) (**E**) are shown. Note that the scale on the y-axis is logarithmic for UCP1 and ADRB3. Lines represent individual paired samples (N = 6) and Ct-values for the reference BAT sample are shown. *P*-values for only statistically significant differences (two sample t test) are shown. *P < 0.05, ***P < 0.001.

Previous reports suggest that murine BMAT would express markers of BAT (16). Moreover, due to its wide distribution in the periphery, BMAT has been suggested to have a role in thermogenesis (5). To test whether BMAT would indeed share metabolic features of brown fat, we studied if GU in BMAT is stimulated by cold exposure. We have previously used similar experimental setting to demonstrate increased GU in human BAT upon cold stimulation (1). We analyzed GU in humeral BMAT, as this could be measured from the same images of the upper body with supraclavicular BAT. In contrast to BAT, cold exposure did not change the glucose utilization in the vertebral or humeral BMAT (Fig. 3A). These data suggest that BMAT in humans does not have a major role in thermogenesis and is metabolically distinct from BAT.

To analyze this in more detail, we collected samples of femoral SAT (Fig. 3B) and femoral and tibial BMAT (Fig. 3C) from healthy, nondiabetic patients during total knee arthroplasty and analyzed the expression of genes characteristic for white, brown, or beige adipocytes. A previously well-characterized BAT sample was used as a reference (1). In line with our PET data, we observed only negligible expression of uncoupling protein 1 (UCP1) and beta3-adrenergic receptor (ADRB3), which are both characteristic for BAT (Fig. 3D). Furthermore, the expression level of PR domain containing 16 (PRDM16), a transcription factor typical for brown adipocytes, was markedly lower in BMAT and SAT samples as compared to the reference BAT sample (Fig. 3D). There was no significant difference in the expression of PRDM16 between BMAT and SAT samples. No significant differences between SAT and BMAT were seen either in the expression of adipocyte markers proliferator-activated receptor gamma (PPARγ) and adiponectin (ADIPOQ), or genes involved in insulin signaling and/or GU, namely insulin receptor (INSR), insulin receptor substrates 1 (IRS1) and 2 (IRS2), and glucose transporter 4 (GLUT4) (Fig. 3E). The expression of T-box protein 1 (TBX1), a marker for beige fat, was lower in BMAT as compared to SAT and the reference BAT sample (Fig. 3D). The expression levels of transmembrane protein 26 (TMEM26) and tumor necrosis factor receptor superfamily member 9 (TNFRSF9 or CD137) were significantly higher in BMAT as compared to SAT but lower as compared to the reference BAT sample (Fig. 3D). The difference in the expression of TMEM26 between BMAT and SAT were confirmed in BMAT samples harvested from pigs that also exhibit high fat accumulation in the marrow space of long bones (data not shown). Based on these data, BMAT is distinct from BAT, as it does not express UCP1, the hallmark of BAT, and GU is not induced by cold exposure. Bone marrow adipose tissue also appears to be molecularly distinct from both SAT and BAT.

## Discussion

Despite its wide prevalence, the function or metabolic activity of BMAT has remained unclear. Traditionally, BMAT was considered an inert filler in the bone marrow, but recently it has been suggested to negatively regulate bone formation, activate bone resorption, serve as an energy reservoir for bone formation, and/or exert systemic effects via secreted factors (7, 29, 30). Studies in mice have implied that BMAT could share some features of BAT based on the expression of markers, such as Dio2 and PGC1α that are enriched in BAT (16). However, in mice the proportion of BMAT in normal mouse long bone is significantly less than in adult humans and the hematopoiesis remains active in the long bone marrow throughout life (6, 17), making mice a suboptimal model to study BMAT.

In this study we demonstrated that human BMAT is a metabolically active, insulin-sensitive tissue. Glucose uptake was observed in femoral BMAT in healthy humans in vivo, and the uptake was significantly increased with insulin stimulation, as evaluated by concurrent hyperinsulinemic euglycemic clamp and [^18^F]FDG-PET imaging. Adult long bone diaphysis is rich in bone marrow adipocytes that indeed comprise an insulin-sensitive fat depot. The trabecular area of vertebral bone had higher basal GU but in contrast to femoral bone, the vertebral GU was not responsive to insulin stimulation. This region of interest contains multiple cell types and the relative contributions of these to the glucose utilization are difficult to discern. Since BM adiposity increases with age, and vertebral GU correlated negatively with age (data not shown), we assumed that the majority of glucose in vertebral bodies is used by other cell types than bone marrow adipocytes, such as hematopoietic cells or bone cells. Interestingly, these cells were not insulin sensitive in our study. Interestingly, we have previously observed that vertebral BM is insulin-responsive in pigs that were first treated with streptozotocin to impair insulin secretion, followed by a high-fat diet (24). In the current study, there even appeared to be a trend towards decreased vertebral GU upon insulin infusion in subjects with T2DM. This discrepancy is likely explained by the severe metabolic challenge of insulin deficiency combined with a high-fat diet in the pig model. Further studies are warranted to elucidate the mechanisms underlying these differences.

Our data also demonstrates that BMAT is not protected from the metabolic consequences of obesity and T2DM. Femur BMAT, which is an insulin-sensitive tissue in healthy, normal-weight subjects, does develop insulin resistance in obesity and T2DM, similarly to other fat depots. Bariatric surgery-induced weight loss significantly improved BMAT GU in obese subjects with and without T2DM, indicating that the insulin sensitivity of BMAT can be restored when the metabolic phenotype is improved. Femur BMAT GU correlated with the whole-body insulin sensitivity (M-value), indicating that it also reflects the overall metabolic status. Thus, other interventions such as conservative weight loss or insulin sensitizing drugs that improve whole body insulin sensitivity most likely also positively affect BMAT. Indeed, we have observed that rosiglitazone, an insulin-sensitizing PPARγ agonist does improve insulin sensitivity in BMAT (unpublished data). Obese subjects with T2DM had elevated inflammatory markers, some of which ameliorated after bariatric surgery (31) together with improvements in BMAT GU. Femur BMAT GU was, however, not associated with inflammation. It only moderately correlated to uCRP in the entire cohort and there was no association between surgery-induced improvements. Thus, our data does not support the concept that the overall inflammatory state or its improvement after weight loss would regulate BMAT GU.

BMAT has been suggested to have a role in thermogenesis, since bone marrow adiposity increases from the periphery to central skeleton with age (17). This has been supported by studies showing the expression of genes characteristic for BAT in mouse long bone samples (16). Nishio et al suggested that cold stimulation would induce GU in vertebra by comparing standardized uptake values (SUVs) under warm and cold conditions (32). However, due to the use of only SUVs instead of properly modeling the quantitative fractional GU into the vertebral tissue, these data are only descriptive. Moreover, the proportion of adipocytes in the vertebral bone marrow is low compared to the long bones. In our study, cold exposure, that was sufficient to activate GU in BAT (1), did not stimulate GU in human long bone BMAT or in the vertebrae in vivo when the PET data was modeled and analyzed using modern methods. In line with the results from PET imaging, we did not observe any significant expression of classical BAT marker genes in BMAT. The gene expression profile of BMAT appeared to be unique and different from white, brown, and beige adipocytes. Glucose uptake of BMAT was not associated with overall bone turnover rate, but it was positively correlated with serum levels of osteocalcin. These findings may suggest that BMAT insulin sensitivity could directly affect osteoblast function, as measured by the production of osteocalcin. Another possibility is that osteocalcin itself could alter the insulin sensitivity of BMAT. The endocortical bone lining cells/osteoblasts are in close proximity to BMAT and osteocalcin has been shown to modulate gene expression and glucose transport in adipocytes in rodents (33, 34).

The question remains what is the contribution of BMAT glucose utilization to the whole-body energy metabolism. We previously measured the amount of fat tissue in multiple fat depots in normal weight and obese subjects using MRI and measured GU in these specific areas with [^18^F]-FDG PET imaging in this same patient cohort (35). Based on the estimate of 3 kg for the BMAT mass, BMAT would constitute approximately 14% of the total fat mass in the normal weight controls in our study. BMAT mass does compare with the amount of abdominal visceral fat (2.2kg) but is less than the amount of abdominal subcutaneous fat (6.8kg). The insulin-stimulated GU in the total visceral fat depot was 44.3 µmol/min in normal-weight subjects (35). Based on the estimated BMAT mass of 3 kg (with tissue density of 0.9196 kg/l) (36), average insulin-stimulated GU in the total BMAT compartment would be approximately 66.6 ± 19.6 µmol/min, that is more than in visceral fat. These data would suggest that BMAT insulin resistance could truly have a clinical significance not only locally but at the systemic level.

The strength of our study is the characterization of human BMAT with multiple approaches, including PET/CT imaging and gene expression analysis. Longitudinal study design in a bariatric surgery study allowed us also to evaluate the changes in BMAT GU in response to weight loss and improvement in insulin sensitivity. One limitation of the study is that due to the resolution of PET, we were only able to assess GU at the tissue level and not at cellular level. Sample size is also limited and future work with larger cohorts are needed to separate sex- and age-specific effects. Also due to the limited number of subjects. we did not perform a subgroup analysis on subjects undergoing sleeve or gastric bypass surgery, although these surgeries can have different physiological effects (25). Also, the gene expression studies were performed on whole tissue, not on isolated adipocytes, and the putative presence of other cell types could not be completely excluded, although our histological analyses showed that the samples were highly enriched in BMAT and almost devoid of other cell types (Fig. 3C). Furthermore, our results apply only to the long bone diaphyseal BMAT, which is considered to contain mainly constitutive BMAT.

In conclusion, BMAT in humans is a molecularly and metabolically distinct fat depot when compared to white, brown, and beige fat. Bone marrow adipose tissue may develop insulin resistance in obesity and T2DM, which can be alleviated by weight loss. Our data demonstrates that BMAT is a metabolically active, insulin-sensitive adipose tissue type that may significantly contribute to the whole body energy metabolism.

## Additional Information

***Disclosure Summary:*** All authors have submitted the ICMJE Form for Disclosure of Potential Conflicts of Interest. Conflicts that the editors consider relevant to the content of the manuscript have been disclosed. The authors have no conflicts of interest relevant to this article.

***Data Availability:*** The datasets generated during and/or analyzed during the current study are not publicly available but are available from the corresponding author on reasonable request.
